# Long non-coding RNA DANCR promotes malignant phenotypes of bladder cancer cells by modulating the miR-149/MSI2 axis as a ceRNA

**DOI:** 10.1186/s13046-018-0921-1

**Published:** 2018-11-12

**Authors:** Yonghao Zhan, Zhicong Chen, Yifan Li, Anbang He, Shiming He, Yanqing Gong, Xuesong Li, Liqun Zhou

**Affiliations:** Department of Urology, Peking University First Hospital, The Institute of Urology, Peking University, National Urological Cancer Centre, No. 8 Xishiku street, Beijing, 100034 China

**Keywords:** DANCR, miR-149, ceRNA, MSI2, Bladder cancer

## Abstract

**Background:**

Accumulating evidences have indicated that long non-coding RNAs (lncRNAs) are potential biomarkers that play key roles in tumor development and progression. Differentiation antagonizing non-protein noding RNA (DANCR) is a novel lncRNA that acts as a potential biomarker and is involved in the development of cancers. However, the clinical significance and molecular mechanism of DANCR in bladder cancer is still unknown.

**Methods:**

The relative expression level of DANCR was determined by Real-Time qPCR in a total of 106 patients with urothelial bladder cancer and in different bladder cancer cell lines. Loss-of-function experiments were performed to investigate the biological roles of DANCR on bladder cancer cell proliferation, migration, invasion and tumorigenicity. Comprehensive transcriptional analysis, RNA-FISH, dual-luciferase reporter assay and western blot were performed to explore the molecular mechanisms underlying the functions of DANCR.

**Results:**

In this study, we found that DANCR was significantly up-regulated in bladder cancer. Moreover, increased DANCR expression was positively correlated with higher histological grade and advanced TNM stage. Further experiments demonstrated that knockdown of DANCR inhibited malignant phenotypes and epithelial-mesenchymal transition (EMT) of bladder cancer cells. Mechanistically, we found that DANCR was distributed mostly in the cytoplasm and DANCR functioned as a miRNA sponge to positively regulate the expression of musashi RNA binding protein 2 (MSI2) through sponging miR-149 and subsequently promoted malignant phenotypes of bladder cancer cells, thus playing an oncogenic role in bladder cancer pathogenesis.

**Conclusion:**

This study is the first to demonstrate that DANCR plays a critical regulatory role in bladder cancer cell and DANCR may serve as a potential diagnostic biomarker and therapeutic target of bladder cancer.

**Electronic supplementary material:**

The online version of this article (10.1186/s13046-018-0921-1) contains supplementary material, which is available to authorized users.

## Background

Urothelial carcinoma of the bladder (BC) is the sixth most common malignancy in men and the most common genitourinary malignancy worldwide; its incidence and mortality have significantly increased over the past decade [1–3]. Although clinical treatments have improved over the past decade, including surgery, radiation therapy, chemotherapy, and immunotherapy, the prognosis of patients diagnosed with BC has not significantly improved [4–6]. The prognosis of bladder cancer is closely related to the stage of disease, but patients do not have specific symptoms at the early stage of bladder cancer [7, 8]. Therefore, finding promising early detection markers and more efficient and safer therapeutic methods have enormous potential significance for improving the clinical strategies and outcomes of bladder cancer.

The long non-coding RNAs (lncRNAs) are an important groups of transcribed RNA molecules that have a length greater than 200 nucleotides [9–11]. The rapid development of RNA genomics has highlighted the role of lncRNAs in many human diseases, especially in cancers [12–15]. Recent accumulating evidences have indicated that lncRNAs, such as SPRY4-IT1, UCA-1, and PANDAR, play important regulatory roles in diverse biological processes in bladder cancer [16–22]. Differentiation antagonizing non-protein noding RNA (DANCR, HGNC:28964) is a novel identified lncRNA located at 4q12.5 [23–26]. Recently, DANCR originally was identified as a potential biomarker and was involved in the development of multiple cancers [27–33]. Although DANCR has been suggested to act as an oncogene, the underlying mechanism by which DANCR-mediated gene expression participates in tumorigenesis remains to be clarified [34, 35]. However, the biological function and underlying mechanism of DANCR in bladder cancer is completely unknown.

In the present study, we showed that DANCR was significantly up-regulated in bladder cancer tissues compared with corresponding non-tumor tissues in a cohort of 106 bladder cancer patients and its expression was significantly correlated with histological grade and TNM stage. Further experiments demonstrated that knockdown of DANCR inhibited malignant phenotypes (proliferation, migration, invasion and EMT and tumorigenicity) of bladder cancer cells. Mechanistically, we found that DANCR was distributed mostly in the cytoplasm and DANCR functioned as a miRNA sponge to positively regulate musashi RNA binding protein 2 (MSI2) expression by sponging miR-149 in a ceRNA-dependent manner. Moreover, knockdown of miR-149 reversed the inhibition of MSI2 expression and MSI2 overexpression reversed the malignant phenotype inhibition of bladder cancer cells induced by silencing DANCR. Together, our results suggest that DANCR is a powerful tumor biomarker, which highlight its potential clinical utility as a promising diagnostic and therapeutic target of bladder cancer.

## Methods

### Patients and clinical samples collection

Between 2011 and 2016, A total of 106 bladder cancer patients who had undergone radical cystectomy without preoperative therapy were enrolled in this study (79 74.5% males; median age 63 years) (Additional file 1: Table S1). Fresh bladder cancer tissue samples and pair-matched normal tissue samples were obtained from patients who underwent radical cystectomy. After resection fresh bladder cancer tissue and pair-matched normal adjacent bladder tissue obtained from the same patient were snap-frozen in liquid nitrogen immediately. All patients included in this study signed informed consent and this study was approved by the Institutional Review Board of Peking University First Hospital, Beijing, China.

### Bladder cancer cell lines and cell culture

Bladder cancer 5637, SW780, UM-UC-3, T24 and SV-HUC-1 cells used in this study were purchased from the Institute of Cell Research, Chinese Academy of Sciences, Shanghai, China. The UM-UC-3, T24 and SV-HUC-1 cells were cultured in Dulbecco’s Modified Eagle Medium (Invitrogen, Carlsbad, CA, USA) plus 10% fetal bovine serum. The SW780 and 5637 cells were cultured in RPMI-1640 Medium (Invitrogen, Carlsbad, CA, USA) plus 10% fetal bovine serum. Corresponding plates were placed at 37 °C with a humidified atmosphere of 5% CO2 in incubator.

### Cell transfection

Plasmid vector PLKO.1-puro and pLVX-EF1α were purchased from BioVector NTCC Inc., Guangzhou, China. The sequences of the related DANCR-shRNA and the negative control were designed and chemically synthesized. These synthetic related sequences were inserted into PLKO.1-puro vector. The sequences of MSI2 and the negative control were chemically synthesized and inserted into pLVX-EF1α vector. The microRNA mimics (agomir) and microRNA inhibitor (antagomir) were purchased from RIBOBIO, Guangzhou, China. Before transfection, the cells were cultured 24 h. Then, the cells were transiently transfected with corresponding vector using Lipofectamine 3000 Transfection Reagent (Invitrogen, Carlsbad, CA, USA) according to the manufacturer’s instructions. After 48 h, cells transfected with corresponding vector were harvested for quantitative real-time PCR. Experiments were repeated at least three times.

### RNA extraction and quantitative real-time PCR

The total RNA of the tissue samples and the transfected cells were extracted using the Trizol reagent (Invitrogen, Carlsbad, CA, USA) according to the manufacturer’s instructions. The detailed primer sequences included in this study are shown in Additional file 2: TableS2. Quantitative real-time PCR was performed using the ABI PRISM 7000 Fluorescent Quantitative PCR System (Applied Biosystems, Foster City, CA, USA) according to the manufacturer’s instructions and normalized to β-actin or U6 small nuclear RNA. Experiments were repeated at least three times.

### Western blotting analysis

Total cell lysates were prepared in a 1× sodium dodecyl sulfate buffer. Total protein was separated by sodium dodecyl sulfate-polyacrylamide gel electrophoresis and transferred onto nitrocellulose membranes. Then the membrane was blocked with 5% non-fat milk and incubated with primary antibodies at 4 °C overnight. After incubation with antibodies specific for MSI2, E-cadherin, N-cadherin, vimentin (Abcam, Hong Kong, China), the blots were incubated with goat anti-rabbit secondary antibody (Abcam, Hong Kong, China) and visualized with enhanced chemiluminescence. Experiments were repeated at least three times.

### Cell counting Kit-8 assay

Cell proliferation was determined using Cell Counting Kit-8 (Beyotime Inst Biotech, China) according to the manufacturer’s instructions. Briefly, 5 × 103 cells/well were seeded in a 96-well flat-bottomed plate, and grown at 37 °C for 24 h, then transfected with corresponding vector. Finally, the absorbance was finally determined at a wavelength of 450 nm using a microplate reader (Bio-Rad, Hercules, CA, USA). Experiments were repeated at least three times.

### Ethynyl-2-deoxyuridine (EdU) incorporation assay

Cell proliferation was also determined by Ethynyl-2-deoxyuridine incorporation assay using an EdU Apollo DNA in vitro kit (RIBOBIO, Guangzhou, China) following the manufacturer’s instructions. Briefly, after transfected with corresponding vector cells were incubated with 100 μl of 50 μM EdU per well for 2 h at 37 °C, respectively. Finally, the cells were visualized under a fluorescence microscopy. Experiments were repeated at least three times.

### Wound healing assay

Cell motility was determined by wound healing assay. At 24 h post transfection, a wound field was created using a sterile 200 μl pipette tip in about 90% confluent cells. The cells were incubated for 24 h at 37 °C, and then the migration of cells was monitored with a digital camera system. The cell migration distance (μm) was calculated by the software program HMIAS-2000. Experiments were repeated at least three times.

### Transwell assay

The invasion of bladder cancer cells was determined using a transwell insert (8 μm, Corning). 24 h after transfection, 5 × 10^4^ cells were first starved in 200 ml serum free medium and then placed in the dishes. The lower chamber was filled with 500 ml of complete medium. The cells were incubated for 48 h at 37 °C, and then the cells that had migrated to the bottom surface of the filter membrane were stained with 0.5% crystal violet solution and photographed. Finally, the absorbance was determined at a wavelength of 570 nm using a microplate reader (Bio-Rad, Hercules, CA, USA). Experiments were repeated at least three times.

### Immunofluorescence

Immunostaining was performed on the paraffin-embedded tumor tissues from nude mice. The avidin-biotin-peroxidase method was adopted to determine the location and relative expression level of the target proteins. The primary antibodies of MSI2, E-cadherin, N-cadherin, vimentin were used at a dilution of 1:2000. Sections were visualized under a fluorescence microscopy (Olympus, Japan). Experiments were repeated at least three times.

### RNA fluorescent in situ hybridization (FISH)

FISH assay was performed using Ribo™ Fluorescent in Situ Hybridization Kit (Ribobio Company, China). DANCR and U6 probes were designed and synthesized by Ribobio Company and labeled with Cy3 fluorescent dye. RNA FISH were performed using fluorescent in situ hybridization kit (RiboBio) following the manufacturer’s instructions. Fluorescence detection was performed with a confocal laser-scanning microscope (Leica, Germany).

### Dual-luciferase reporter assay

MSI2-WT/MUT (GenePharma, Shanghai, China) were constructed and transfected into T24 along with Agomir-149b/Agomir-NC. Luciferase activity was detected using the Dual-Luciferase Reporter Assay System (Promega; 48 h after transfection) according to the manufacturer’s instructions. Experiments were repeated at least three times.

### Tumor xenograft implantation in nude mice

Animal work was permitted by the Institutional Animal Care and Use Committee (IACUC) of Peking University First Hospital (Beijing, China), and conducted in accordance with its recommendations and ethical regulations. The mice were maintained under standard conditions according to the institutional guidelines for animal care. BCCs were collected after transfection for 48 h. 5 × 10^5^ BCCs were injected subcutaneously into BALB/c-Nude mice. The mice were euthanized after 8 weeks.

### Statistical analyses

All experimental data from three independent experiments were analyzed by Student’s t-test or χ2 test and results were expressed as mean ± standard deviation. *P*-values of less than 0.05 were considered to be statistically significant. All statistical tests were conducted by SPSS version 19.0 software (SPSS Inc. Chicago, IL, USA).

## Results

### DANCR expression is up-regulated in bladder cancer tissues and cell lines

**Fig. 1:**
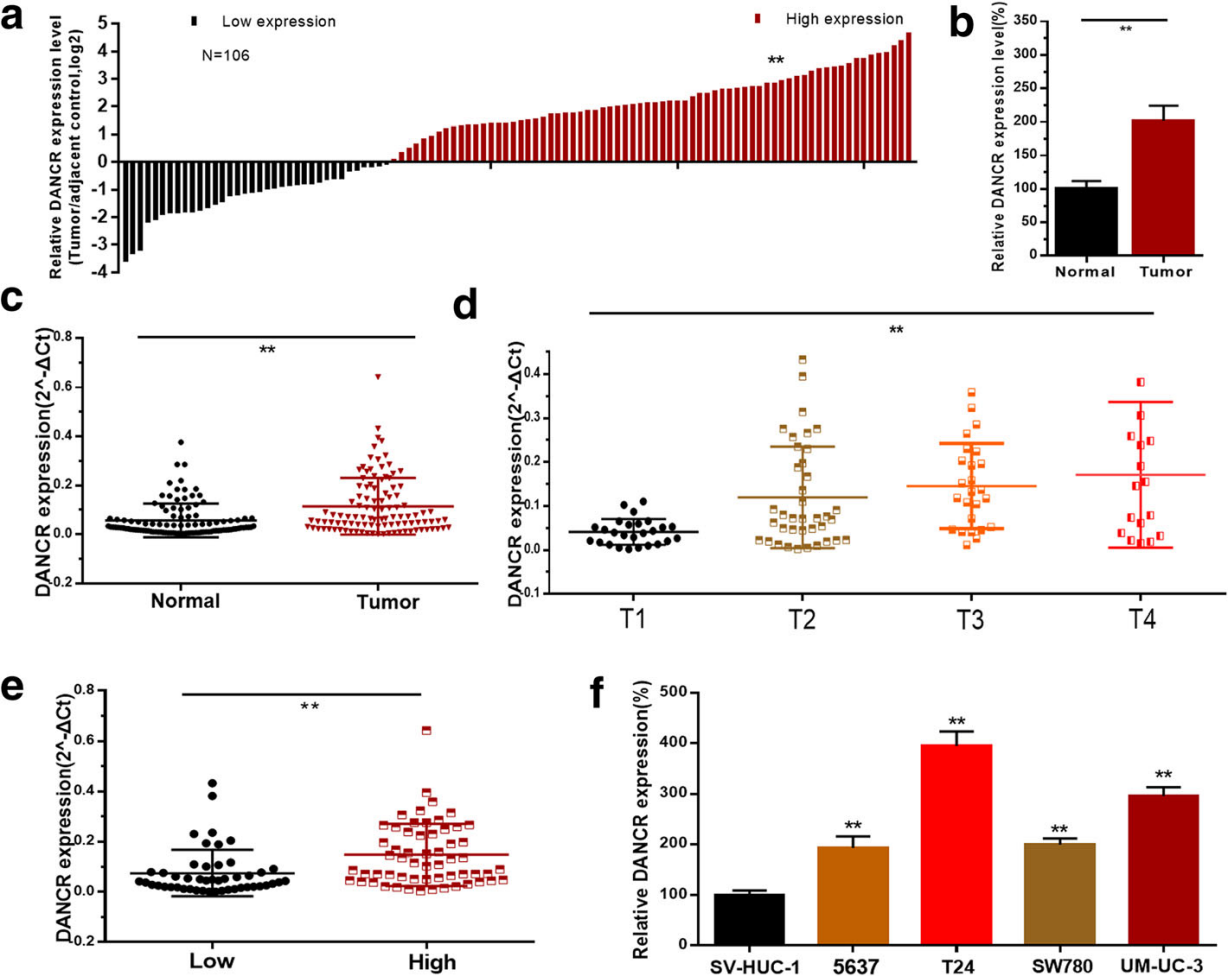
The relative expression levels of DANCR in bladder cancer. **a**: The heights of the columns in the chart represent the log2-transformed fold changes (bladder cancer tissue/normal bladder tissue) in DANCR expression in 106 patients with bladder cancer. **b** and **c**: DANCR is up-regulated in bladder cancer tissues compared with corresponding non-tumor tissues. **d**: DANCR is up-regulated in bladder cancer patients with advanced TNM stage. **e**: DANCR is up-regulated in bladder cancer patients with higher histological grade. **f**: DANCR is up-regulated in bladder cancer cell lines compared to normal urothelial cell line. Data are shown as mean ± SD. *p < 0.05; **p < 0.01

The relative expression level of DANCR was determined by qRT-PCR in bladder cancer tissues and cell lines. The CASC9 expression fold change (bladder cancer tissue/matched normal tissue) in each patient was indicated in Fig. 1a. DANCR expression was up-regulated in 66.05% (70/106) of bladder cancer tissues compared with the corresponding normal tissue samples (Fig. 1b, c). Moreover, elevated DANCR expression was positively correlated with advanced TNM stage (Fig. 1d) and higher histological grade (Fig. 1e). DANCR expression was up-regulated in BC cell lines compared with the normal urothelial cell line SV-HUC-1 (Fig. 1f). Clinicopathological features of patients and statistical results are shown in Table 1 and Additional file 1: Table S1, respectively.

**Table 1:** Correlation between DANCR expression and clinicopathological features of UCB patients

Parameters Total	Group	Total	DANCR expression		*P* value
			High	Low	
Gender	Male	79 (75%)	55 (52%)	24 (23%)	0.183
	Female	27 (25%)	15 (14%)	12 (11%)	
Age (years)	< 60	37 (35%)	25 (24%)	12 (11%)	0.808
	≥ 60	69 (65%)	45 (42%)	24 (23%)	
Tumor size (cm)	< 3 cm	42 (40%)	26 (25%)	16 (15%)	0.467
	≥ 3 cm	64 (60%)	44 (42%)	20 (18%)	
Multiplicity	Single	59 (56%)	37 (35%)	22 (21%)	0.418
	Multiple	47 (44%)	33 (31%)	14 (13%)	
Histological grade	L	48 (46%)	25 (24%)	23 (22%)	0.006*
	H	58 (54%)	45 (42%)	13 (12%)	
Tumor stage T	Ta,T1	26 (24%)	11 (10%)	15 (14%)	0.003*
	T2-T4	80 (76%)	59 (56%)	21 (20%)	
Lymph nodes metastasis	NO	92 (87%)	59 (56%)	33 (31%)	0.447
	YES	14 (13%)	11 (10%)	3 (3%)	

*P < 0.05 was considered significant (Chi-square test between 2 groups)

### Knockdown of DANCR inhibits cell proliferation of bladder cancer cells

**Fig. 2:**
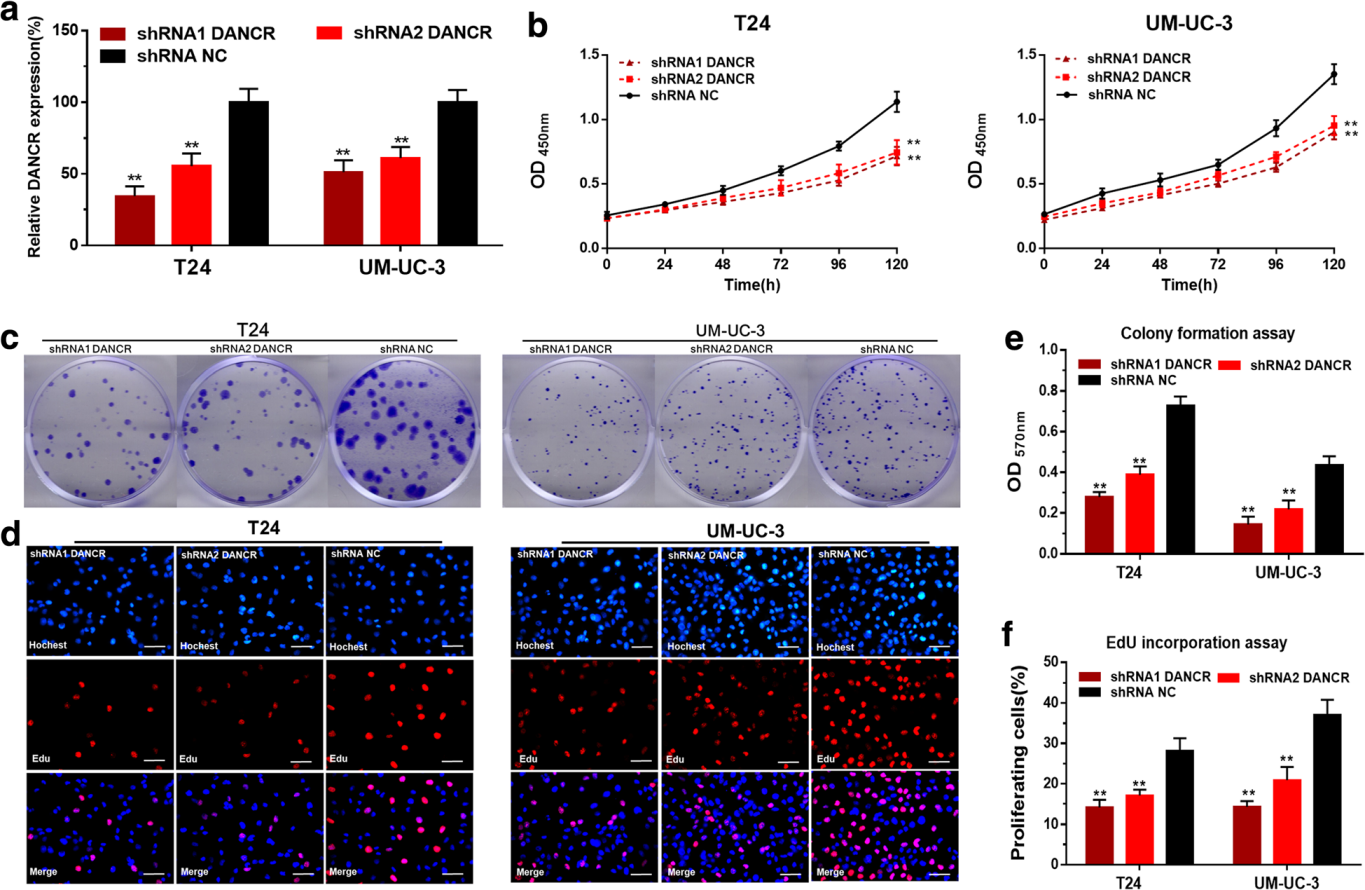
The effect of DANCR on cell proliferation of bladder cancer cells. **a**: The DANCR specific shRNAs significantly decreased the expression level of DANCR in T24 and UM-UC-3. **b**: The cell proliferation changes of bladder cancer cells were determined using CCK-8 assay. **c** and **e**: The cell proliferation changes of bladder cancer cells were determined using colony-formation assay. Inhibited cell proliferation by silencing DANCR was observed in T24 and UM-UC-3. **d** and **f**: The cell proliferation changes of bladder cancer cells were determined using Edu assay. Inhibited cell proliferation by silencing DANCR was observed in T24 and UM-UC-3. Data are shown as mean ± SD. *p < 0.05; **p < 0.01

We further determined whether DANCR regulated cell proliferation of bladder cancer cells. The DANCR specific shRNAs significantly down-regulated the expression level of DANCR in T24 and UM-UC-3 cells (Fig. 2a). The cell proliferation changes of bladder cancer cells were determined using CCK-8 assay, colony-formation assays and Edu assay. Inhibited cell proliferations were both observed in T24 and UM-UC-3 cells by silencing DANCR (Fig. 2b-f). These results demonstrated that DANCR promotes cell proliferation of bladder cancer cells.

### Knockdown of DANCR inhibits cell migration, invasion and EMT of bladder cancer cells

**Fig. 3:**
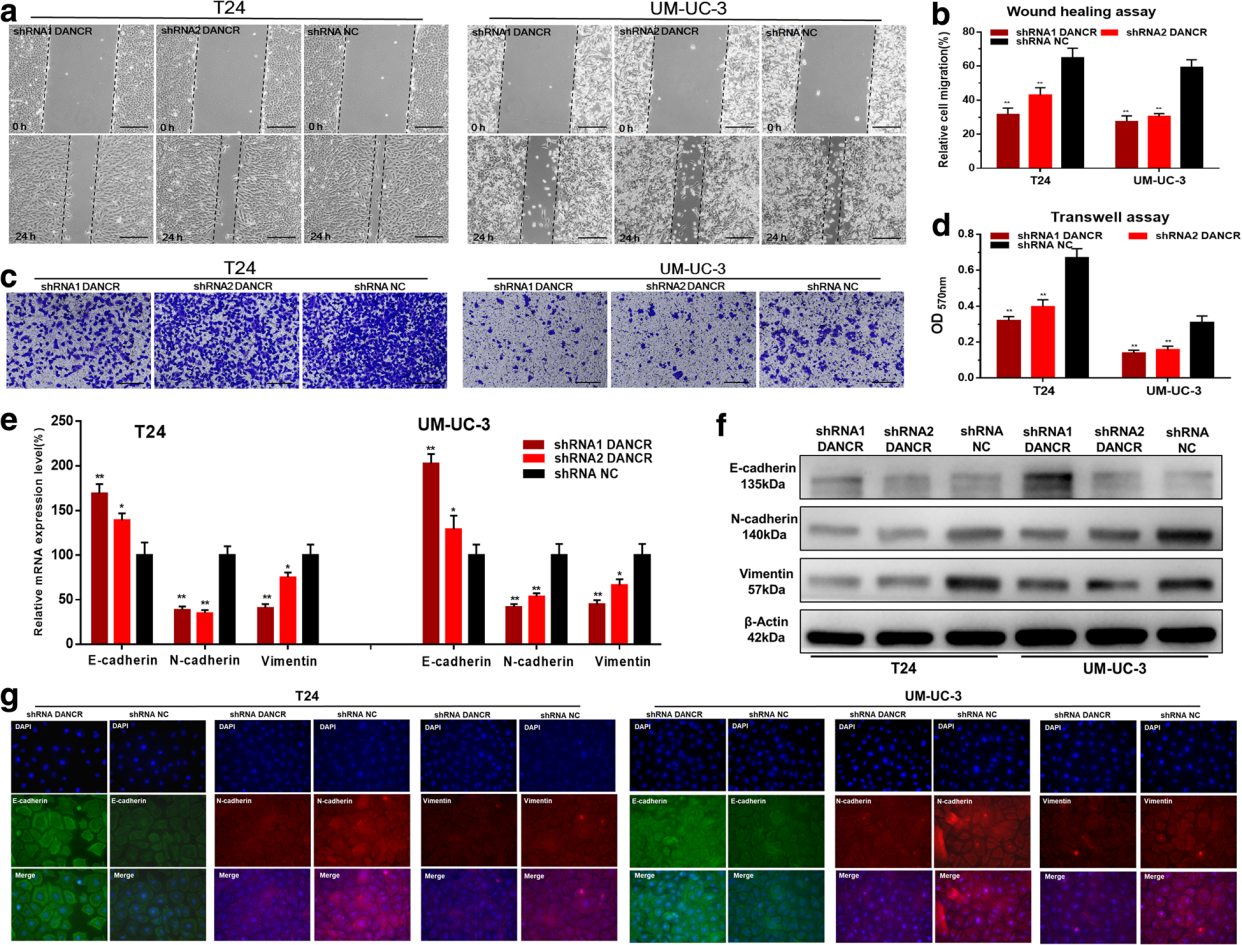
The effect of DANCR on migration, invasion and EMT of bladder cancer cells. **a** and **b**: The migratory abilities of bladder cancer cells were determined using wound healing assay. Inhibited cell migration by silencing DANCR was observed in T24 and UM-UC-3. **c** and **d**: The invasive abilities of bladder cancer cells were determined using transwell assay. Inhibited cell invasion by silencing DANCR was observed in T24 and UM-UC-3. **e**-**g**: The expression of EMT markers were determined using qRT-PCR, western blotting and immunofluorescence. Knockdown of DANCR increased the expression of E-cadherin and decreased the expression of N-cadherin and vimentin in bladder cancer cells. Data are shown as mean ± SD. *p < 0.05; **p < 0.01

We further determined whether DANCR regulated cell migration and invasion of bladder cancer cells. The migratory abilities of bladder cancer cells were determined using wound healing assay. Inhibited cell migrations were observed in T24 and UM-UC-3 induced by silencing DANCR (Fig. 3a, b). The invasive abilities of bladder cancer cells were determined using transwell assay. Inhibited cell invasions were observed in T24 and UM-UC-3 induced by silencing DANCR (Fig. 3c, d). We further determined whether DANCR regulated EMT of bladder cancer cells. The expression of EMT markers were determined using qRT-PCR, western blotting and immunofluorescence. Knockdown of DANCR increased E-cadherin expression and decreased N-cadherin and vimentin expression in bladder cancer cells (Fig. 3e, f, g). The results indicated that DANCR promotes cell migration, invasion and EMT of bladder cancer cells.

### DANCR positively regulates MSI2 expression via sponging miR-149

**Fig. 4:**
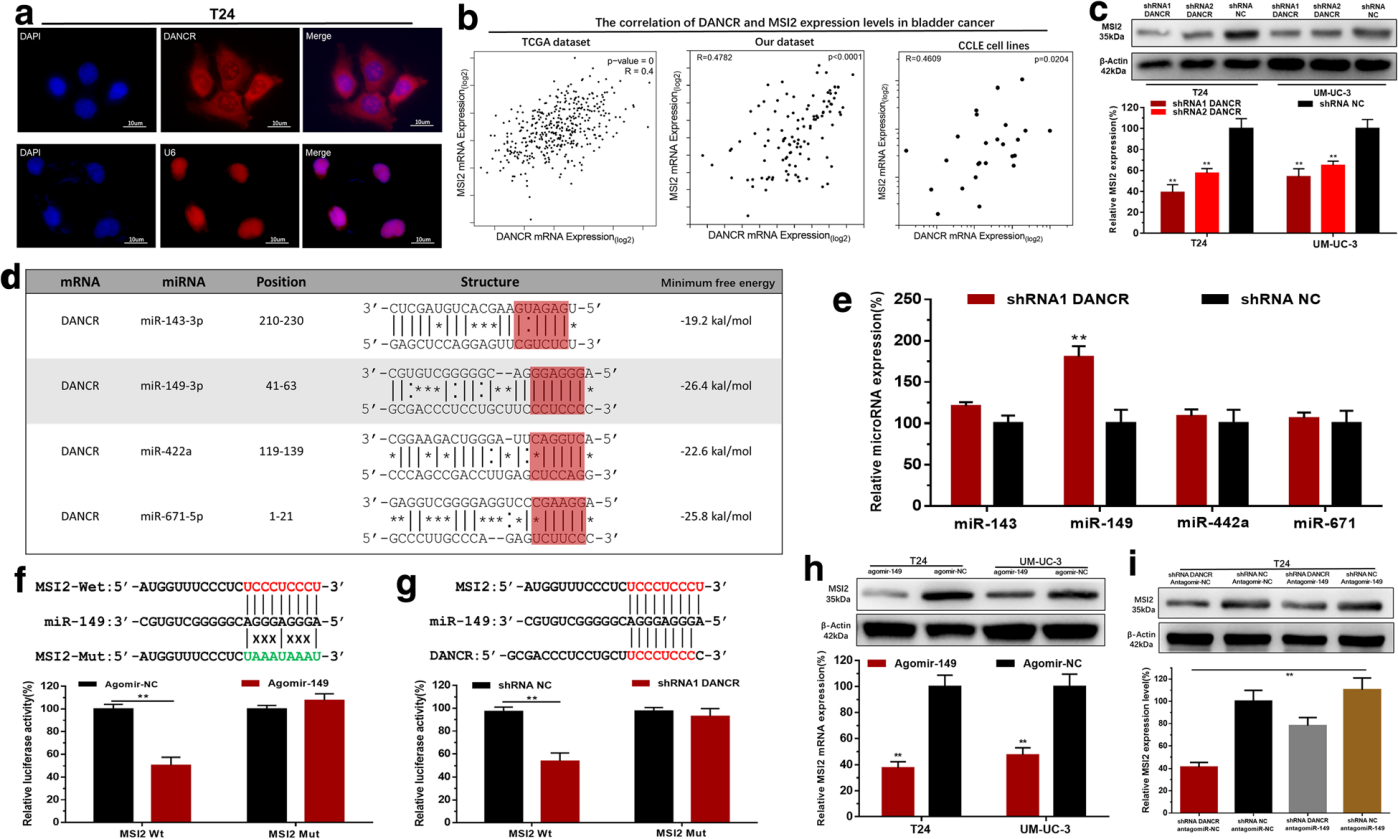
DANCR positively regulates MSI2 expression via sponging miR-149. **a**: The RNA-FISH results revealed that DANCR was distributed mostly in the cytoplasm in bladder cancer cells. **b**: The comprehensive transcriptional analysis results showed that DANCR expression levels were statistically positively correlated with MSI2 expression levels in bladder cancer. **c**: Knockdown of DANCR decreased MSI2 expression in bladder cancer cells. **d**: The bio-information analysis results showed that DANCR and MSI2 have common putative binding sites with multiple miRNAs. **e**: Knockdown of DANCR increased miR-149 expression in bladder cancer cells. **f**: Bioinformatics analysis showed that the 3’UTR sequence of MSI2 is complementary to the seed sequence of miR-149. Dual-luciferase reporter assay showed MSI2-Wt and Agomir149 co-transfection significantly inhibited luciferase activity. **g**: Knockdown of DANCR decreased the luciferase activity of bladder cancer cells transfected with MSI2-Wt. **h**: Overexpressing miR-149 decreased the expression of MSI2 in bladder cancer cells. **i**: Knockdown of miR-149 increased MSI2 expression in bladder cancer cells transfected with shRNA-DANCR. Data are shown as mean ± SD. *p < 0.05; **p < 0.01

The subcellular localization of lncRNAs is closely related to their biological function and potential molecular roles. First, we detected the subcellular localization of DANCR using RNA-FISH. The results showed that most of the positives orientated in the cytoplasm, minority in the nucleus (Fig. 4a). To investigate the underlying mechanisms of DANCR-mediated biological processes, we performed qRT-PCR and comprehensive transcriptional analysis by using TCGA dataset, CCLE dataset and our dataset. The results showed that DANCR expression levels were statistically positively correlated with MSI2 expression levels in bladder cancer (Fig. 4b) and knockdown of DANCR decreased MSI2 expression in bladder cancer cells (Fig. 4c). Through searching online bioinformatics database, bio-information analysis predicted that DANCR and MSI2 had common putative binding sites with multiple miRNAs (Fig. 4d). Detailed prediction results were shown in Additional file 3: Table S3. Then we found knockdown of DANCR increased miR-149 expression in bladder cancer cells (Fig. 4e). Bioinformatics analysis showed that the 3’UTR sequence of MSI2 was complementary to the seed sequence of miR-149 (Fig. 4f). Dual-luciferase reporter assay showed MSI2-Wt and Agomir149 co-transfection significantly inhibited luciferase activity, and MSI2-Mut and Agomir149 co-transfection failed to change luciferase activity (Fig. 4f). Furthermore, knockdown of DANCR decreased the luciferase activity of cells transfected with MSI2-Wt (Fig. 4g). We further determined whether DANCR regulated the expression of MSI2 in BC cells via miR-149-dependent manner. We found overexpressing miR-149 decreased MSI2 expression in BC cells (Fig. 4h). Moreover, knockdown of miR-149 reversed the MSI2 expression inhibition of BC cells induced by silencing DANCR (Fig. 4i). The results indicated that DANCR positively regulates MSI2 expression via sponging miR-149 in BC cells.

### Overexpressing of MSI2 reverses malignant phenotypes inhibition of bladder cancer cells induced by silencing DANCR

**Fig. 5:**
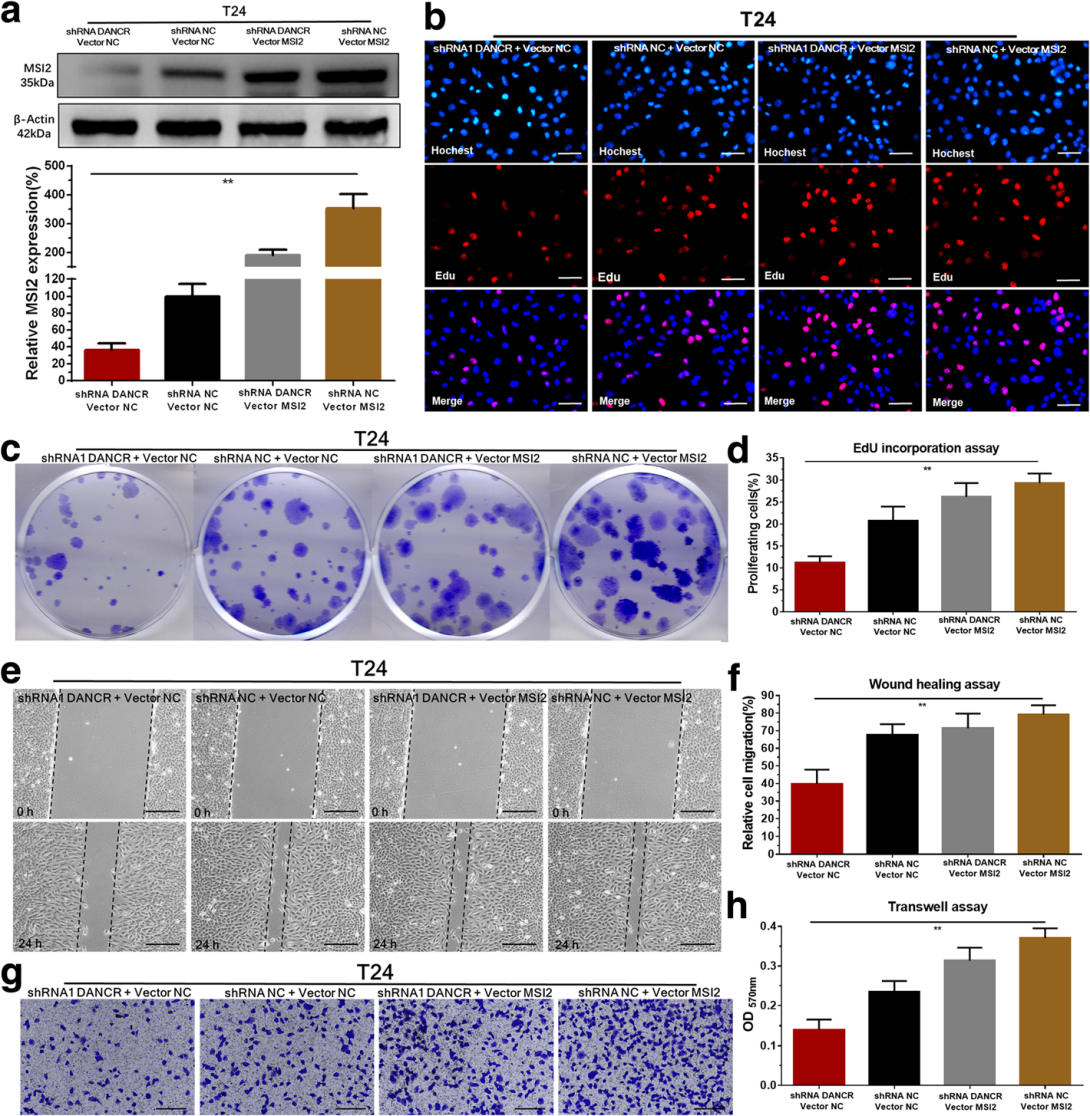
Overexpressing of MSI2 reversed malignant phenotypes inhibition of bladder cancer cells induced by silencing DANCR. **a**: The MSI2 specific vector significantly reversed MSI2 expression inhibition induced by silencing DANCR in bladder cancer cells. **b**-**d**: Overexpressing MSI2 significantly reversed cell proliferation inhibition induced by silencing DANCR. **e** and **f**: Overexpressing MSI2 significantly reversed cell migration inhibition induced by silencing DANCR. **g** and **h**: Overexpressing MSI2 significantly reversed cell invasion inhibition induced by silencing DANCR. Data are shown as mean ± SD. *p < 0.05; **p < 0.01

We further determined whether DANCR regulated malignant phenotypes of bladder cancer cells via MSI2-dependent manner. Our results showed that the MSI2 specific vector (Plvx-MSI2) significantly reversed the inhibition of MSI2 expression induced by silencing DANCR in BC cells (Fig. 5a). Meanwhile, we found MSI2 overexpression significantly reversed cell proliferation inhibition of BC cells (Fig. 5b, c, d) induced by silencing DANCR. Moreover, MSI2 overexpression significantly reversed cell migration (Fig. 5e, f) and invasion (Fig. 5g, h) inhibition of bladder cancer cells induced by silencing DANCR. The results indicated that DANCR promotes malignant phenotypes of bladder cancer cells via MSI2-dependent manner.

### Knockdown of DANCR inhibits tumorigenicity of bladder cancer cells

**Fig. 6:**
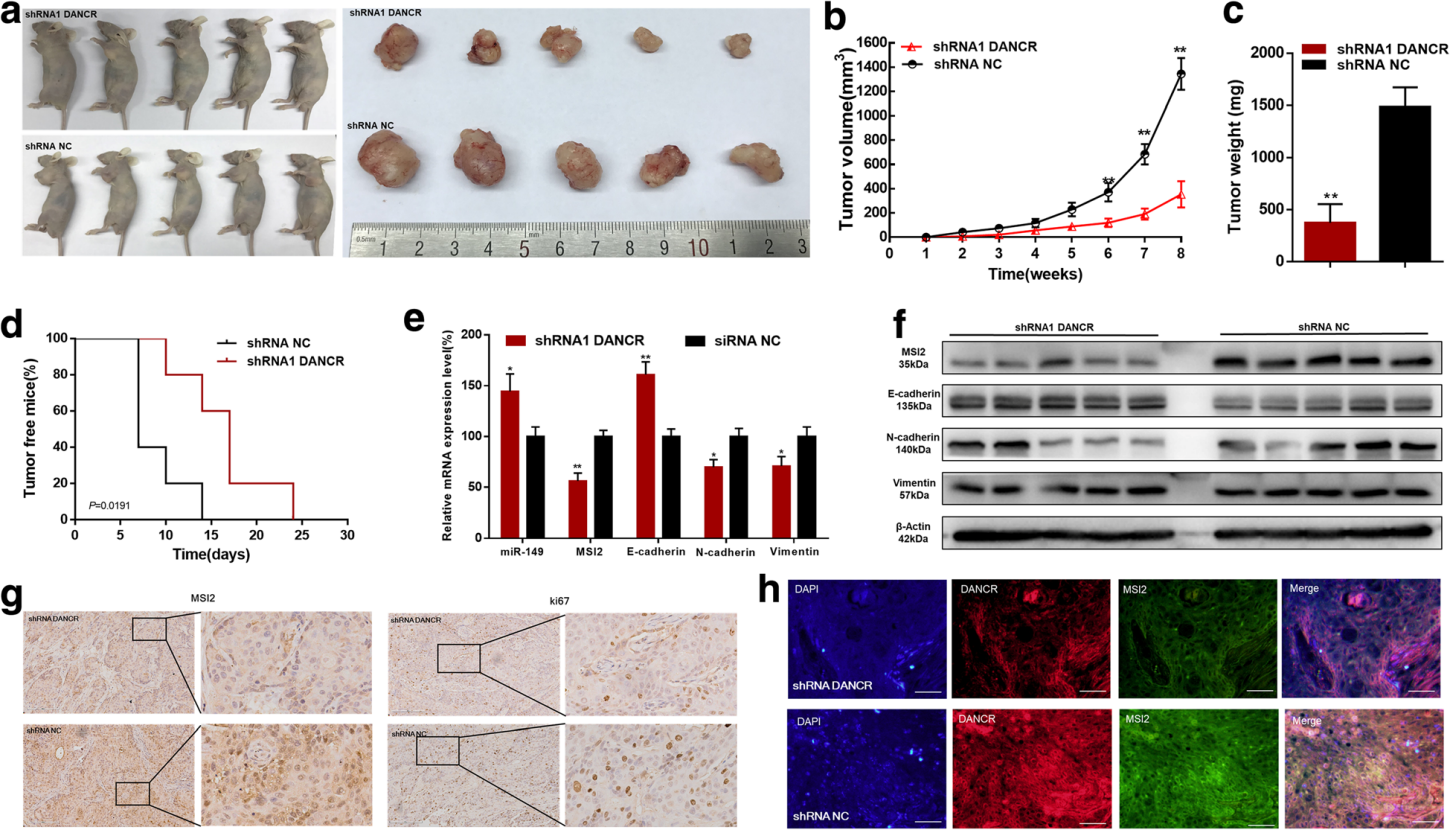
The effect of DANCR on tumorigenicity of bladder cancer cells. **a**: Tumors collected from mice were exhibited. **b**: Tumor volume curve of DANCR or NC treatment groups were measured and analyzed. **c**: Tumor free mouse proportion of DANCR or NC treatment groups were measured and analyzed. **d**: Tumor weight of DANCR or NC treatment groups were measured and analyzed. Knockdown of DANCR inhibited the tumorigenicity of bladder cancer cells in vivo. **e** and **f**: Knockdown of DANCR increased miR-149 expression, decreased MSI2 expression and inhibited EMT of bladder cancer cells in vivo. **g**: Knockdown of DANCR decreased MSI2 and Ki67 expression of bladder cancer cells in vivo. **h**: Knockdown of DANCR decreased MSI2 expression of bladder cancer cells. Moreover, DANCR and MSI2 were co-localized in bladder cancer cells in vivo. Data are shown as mean ± SD. *p < 0.05; **p < 0.01

**Fig. 7:**
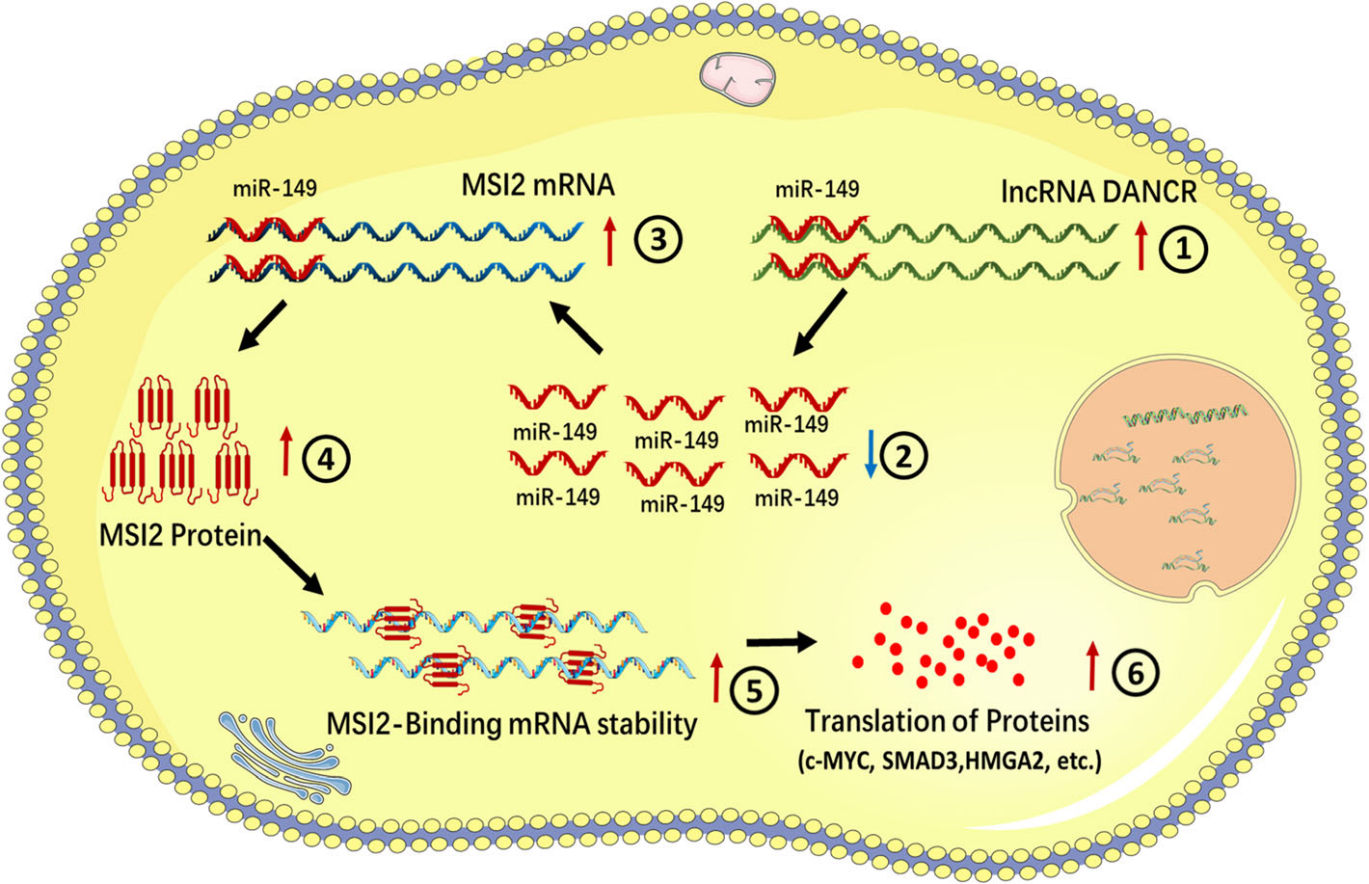
The schematic diagram of the oncogenic role of DANCR in bladder cancer cells. DANCR functions as a miRNA sponge to positively regulate MSI2 expression through sponging miR-149 and subsequently promotes malignant phenotypes of bladder cancer cells, thus playing an oncogenic role in bladder cancer pathogenesis

We further determined whether DANCR regulated tumorigenicity of BC cells using generation of xenograft. We found knockdown of DANCR inhibited the tumorigenicity of bladder cancer cells in vivo (Fig. 6a-d). Tumors collected from mice were exhibited and measured (Fig. 6a). Tumor growth of NC treatment group was faster than that in the shDANCR group (Fig. 6b). Tumor weight of NC treatment group was greater than that in the shDANCR group (Fig. 6c). Tumor free mouse proportion of shDANCR group was higher than that in the NC treatment group (Fig. 6d). We found knockdown of DANCR decreased MSI2 expression and inhibited EMT of bladder cancer cells in vivo (Fig. 6e, f). Meanwhile, we found knockdown of DANCR inhibited MSI2 and ki67 expression (Fig. 6g) of bladder cancer cells in vivo. Moreover, we found that DANCR and MSI2 were co-localized in bladder cancer cells (Fig. 6h). The results indicated that DANCR promoted tumorigenicity of bladder cancer cells via up-regulating MSI2. As shown in Fig. 7, we found that DANCR was significantly up-regulated in bladder cancer cells and DANCR functioned as a miRNA sponge to positively regulate MSI2 expression through sponging miR-149. Elevated MSI2 protein promoted transcription and translation of proteins operating in essential oncogenic signaling pathways, subsequently promoting malignant phenotypes of bladder cancer cells.

## Discussion

Bladder cancer is the most common genitourinary malignancies all over the world [36, 37]. There are no specific symptoms for patients with bladder cancer at the early stage, therefore, some bladder cancers are diagnosed when treatments are less effective [38–40]. Therefore, finding new prognostic and therapeutic target have enormous potential significance to improving the clinical strategies and outcomes of bladder cancer.

The lncRNAs are important new members of non-coding RNA family, which are longer than 200 nucleotides [41, 42]. Recently, an increasing number of evidences have indicated that lncRNAs regulate gene expression at different processing levels, including chromatin modification, transcription and posttranscriptional regulation [43, 44]. For example, HOTAIR and Xist have also been reported to interact with the chromatin remodeling protein PRC2 complex to repress gene regulation [45, 46]. MALAT1 and ATB have also been reported to downregulate the expression of miR-200c and subsequently increase the expression of ZEB2/ZNF217 in a ceRNA-dependent manner [47, 48]. DANCR is a newly identified lncRNA mapped to human chromosome 4q12.5 locus [49]. Recent studies have provided evidence that DANCR plays a key role in transcription regulation of targeted gene and the disregulation of DANCR expression have become highlighted in some somatic cancers [50–55]. For example, DANCR can interact with the binding site of miR-199a/320a/214 in 3’UTR of CTNNB1 to inhibit the suppression of CTNNB1 by miRNAs, thus promoting stemness features of hepatocellular carcinoma [29]. DANCR competitively binds to miR-335-5p and miR-1972 to regulate the expression of ROCK1, thus promoting the malignant biological behaviours of osteosarcoma [56]. However, the clinical significance and biological function of DANCR in bladder cancer are completely unknown.

To the best of our knowledge, this is the first report of DANCR being involved in the development of bladder cancer. In this study, we found the expression level of DANCR was significantly up-regulated in bladder cancer tissues compared with corresponding non-tumor tissues and increased DANCR expression was positively correlated with higher histological grade and advanced TNM stage. Meanwhile, the expression level of DANCR was significantly up-regulated in bladder cancer cell lines compared with normal urothelial cell line. Further experiments demonstrated that knockdown of DANCR inhibited malignant phenotypes (proliferation, migration, invasion, EMT and tumorigenicity) of bladder cancer cells. Mechanistically, we found that DANCR expression was statistically positively correlated with MSI2 expression in bladder cancer and knockdown of DANCR decreased MSI2 expression in bladder cancer cells. MSI2 can bind and regulate the mRNA stability and translation of proteins operating in essential oncogenic signaling pathways, such as NUMB/Notch, PTEN/mTOR, TGF-β/SMAD3, MYC, cMET [57]. Based on these biological functions, MSI2 protein can regulate cancer invasion, metastasis and development of more aggressive cancer phenotypes, including drug and radiation resistance [58]. LncRNA subcellular localization patterns can provide fundamental insights into their biology and fosters hypotheses for potential molecular roles. Then we detected the subcellular location of DANCR using RNA-FISH. RNA-FISH results revealed that DANCR was distributed mostly in the cytoplasm, suggesting that DANCR might also play a role in posttranscriptional level in bladder cancer. Through searching online bioinformatics database, bio-information analysis predicted that DANCR and MSI2 have common putative binding sites with miR-149. Further experiments demonstrated that knockdown of DANCR increased miR-149 expression and subsequently inhibited the expression of MSI2 in a ceRNA-dependent manner. Moreover, knockdown of miR-149 reversed MSI2 expression and MSI2 overexpression reversed the malignant phenotype inhibition of bladder cancer cells induced by silencing DANCR.

## Conclusions

Our study reveals that DANCR functions as a miRNA sponge to positively regulate MSI2 expression through sponging miR-149 and subsequently promotes the malignant phenotypes of bladder cancer cells, thus playing an oncogenic role in bladder cancer pathogenesis. The results of this study provide a new basis for studying the mechanism of the occurrence and development of bladder cancer. Cumulatively, our results suggest that DANCR is a powerful tumor biomarker, which highlight its potential clinical utility as a promising diagnostic and therapeutic target of bladder cancer.

## Additional files

Additional file 1: **Table S1.** Summary of clinicopathological features of tissues of bladder cancer. (DOCX 19 kb)

Additional file 2: **Table2.** The primer sequences included in this study. (DOCX 16 kb)

Additional file 3: **Table S3.** Results of bioinformation analysis. (DOCX 17 kb)

## Abbreviations

BC: Bladder cancer
CCLE: Cancer cell line encyclopedia
ceRNA: Competing endogenous RNA
DANCR: Differentiation antagonizing non-protein noding RNA
EdU: Ethynyl-2-deoxyuridine
EMT: Epithelial-mesenchymal transition
FISH: Fluorescent in situ hybridization
lncRNA: Long non-coding RNA
MSI2: Musashi RNA Binding Protein 2
qRT-PCR: Quantitative real time-PCR
TCGA: The Cancer Genome Atlas
