# Correction: Long non-coding RNA DANCR promotes malignant phenotypes of bladder cancer cells by modulating the miR-149/MSI2 axis as a ceRNA

**DOI:** 10.1186/s13046-023-02736-8

**Published:** 2023-07-01

**Authors:** Yonghao Zhan, Zhicong Chen, Yifan Li, Anbang He, Shiming He, Yanqing Gong, Xuesong Li, Liqun Zhou

**Affiliations:** Department of Urology, Peking University First Hospital, The Institute of Urology, Peking University, National Urological Cancer Centre, No. 8 Xishiku Street, Beijing, 100034 China

**Correction:** ***J Exp Clin Cancer Res*** **37, 273 (2018)**

**https://doi.org/10.1186/s13046-018-0921-1**

Following publication of the original article [1], the authors identified an error in the images of Fig. 5, specifically:

• Figure 5e - the 24 h wound healing of shRNA1 DANCR + Vector MSI2 group was misplaced

The correct figure is given below.

**Fig. 5:**
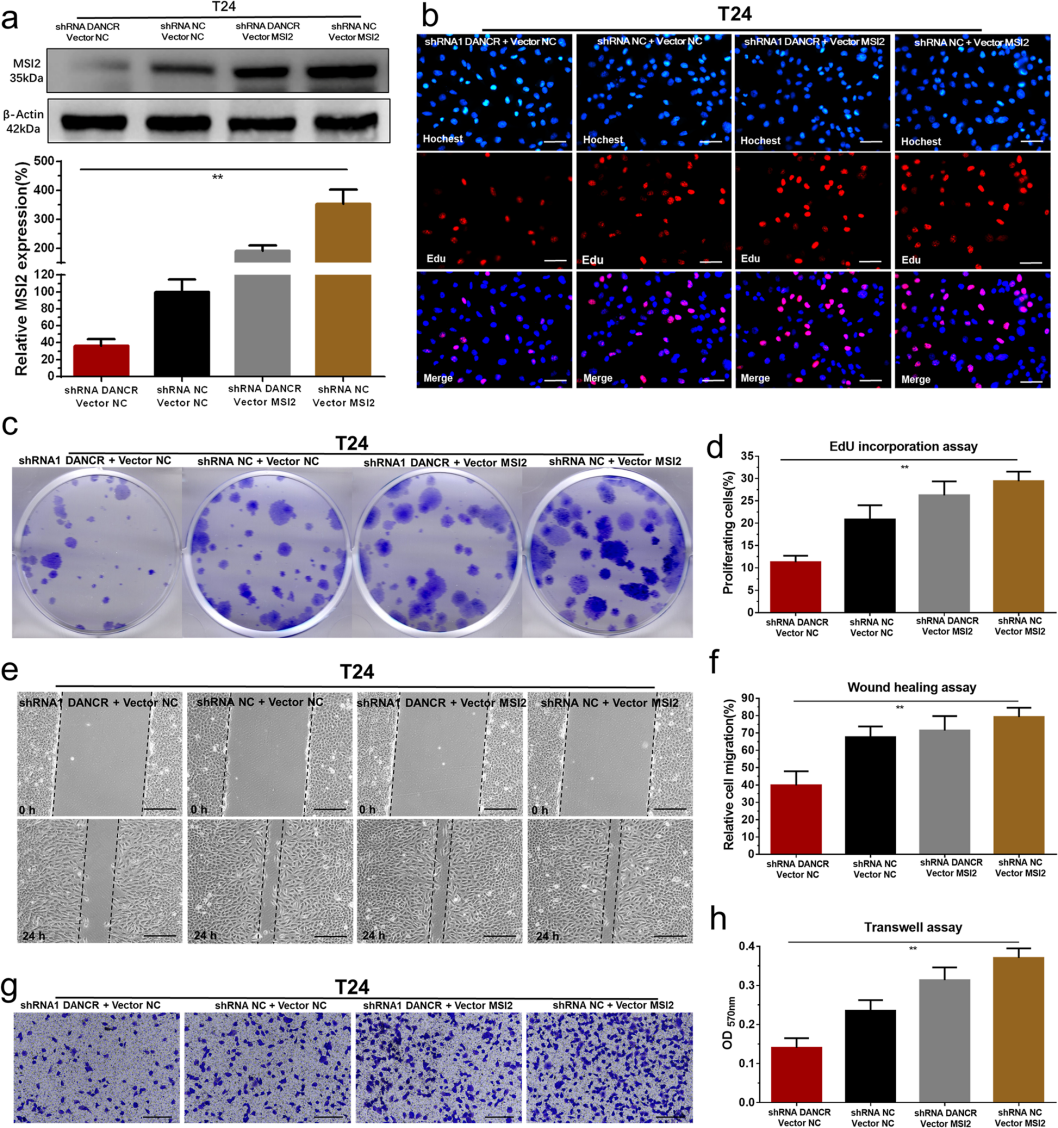
Overexpressing of MSI2 reversed malignant phenotypes inhibition of bladder cancer cells induced by silencing DANCR. **a** The MSI2 specific vector significantly reversed MSI2 expression inhibition induced by silencing DANCR in bladder cancer cells. **b-d** Overexpressing MSI2 significantly reversed cell proliferation inhibition induced by silencing DANCR. **e** and **f** Overexpressing MSI2 significantly reversed cell migration inhibition induced by silencing DANCR. **g** and **h** Overexpressing MSI2 significantly reversed cell invasion inhibition induced by silencing DANCR. Data are shown as mean ± SD. *p < 0.05; **p < 0.01
