# Supplementary material for: Long non-coding RNA DANCR promotes malignant phenotypes of bladder cancer cells by modulating the miR-149/MSI2 axis as a ceRNA
Source: J Exp Clin Cancer Res. 2018 Nov 12;37:273. doi: 10.1186/s13046-018-0921-1 (PMC6233575; doi:10.1186/s13046-018-0921-1)
Supplement: Supplementary file 2 — Table2. The primer sequences included in this study. (DOCX 16 kb) [file 13046_2018_921_MOESM2_ESM.docx]

**Table S2. The primer sequences included in this study.**

| **Gene** | **Accession number** |  | **Primer sequences(5’-3’)** |
| --- | --- | --- | --- |
| DANCR | HGNC:28964 | Forward | GCGCCACTATGTAGCGGGTT |
|  |  | Reverse | TCAATGGCTTGTGCCTGTAGTT |
| MSI2 | HGNC:18585 | Forward | ACCTCACCAGATAGCCTTAGAG |
|  |  | Reverse | AGCGTTTCGTAGTGGGATCTC |
| E-cadherin | HGNC: 1748 | Forward | ATTTTTCCCTCGACACCCGAT |
|  |  | Reverse | TCCCAGGCGTAGACCAAGA |
| N-cadherin | HGNC: 1759 | Forward | AGCCAACCTTAACTGAGGAGT |
|  |  | Reverse | GGCAAGTTGATTGGAGGGATG |
| Vimentin | HGNC: 12692 | Forward | AGTCCACTGAGTACCGGAGAC |
|  |  | Reverse | CATTTCACGCATCTGGCGTTC |
| MiR-149-3P | MIMAT0004609 | Forward | AGGGAGGGACGGGGGCT |
| U6 |  | Forward | CTCGCTTCGGCAGCACA |
|  |  | Reverse | AACGCTTCACGAATTTGCGT |
| β-actin | HGNC: 132 | Forward | GCGGACTATGACTTAGTTGCGTTACA |
|  |  | Reverse | TGCTGTCACCTTCACCGTTCCA |
