# Supplementary material for: Long non-coding RNA DANCR promotes malignant phenotypes of bladder cancer cells by modulating the miR-149/MSI2 axis as a ceRNA
Source: J Exp Clin Cancer Res. 2018 Nov 12;37:273. doi: 10.1186/s13046-018-0921-1 (PMC6233575; doi:10.1186/s13046-018-0921-1)
Supplement: Supplementary file 3 — Table S3. Results of bioinformation analysis. (DOCX 17 kb) [file 13046_2018_921_MOESM3_ESM.docx]

**Table S3. Results of bioinformation analysis.**

| **microRNAs** | **lncRNA** | **mRNA** |
| --- | --- | --- |
| hsa-miR-143-3p | DANCR | MSI2 |
| hsa-miR-149-3p | DANCR | MSI2 |
| hsa-miR-422a | DANCR | MSI2 |
| hsa-miR-596 | DANCR | MSI2 |
| hsa-miR-671-5p | DANCR | MSI2 |
| hsa-miR-1207-5p | DANCR | MSI2 |
| hsa-miR-1321 | DANCR | MSI2 |
| hsa-miR-2467-5p | DANCR | MSI2 |
| hsa-miR-4271 | DANCR | MSI2 |
| hsa-miR-4649-3p | DANCR | MSI2 |
| hsa-miR-4695-5p | DANCR | MSI2 |
| hsa-miR-4725-3p | DANCR | MSI2 |
| hsa-miR-4728-5p | DANCR | MSI2 |
| hsa-miR-4731-5p | DANCR | MSI2 |
| hsa-miR-4739 | DANCR | MSI2 |
| hsa-miR-4756-5p | DANCR | MSI2 |
| hsa-miR-4763-3p | DANCR | MSI2 |
| hsa-miR-5196-5p | DANCR | MSI2 |
